# Electrical impedance tomography: the solution for lung morphology assessment?

**DOI:** 10.1186/s13613-020-00719-y

**Published:** 2020-08-03

**Authors:** Florian Blanchard, Adrien Picod, Jean-Michel Constantin

**Affiliations:** 1 Medecine Sorbonne-Université, DMU DREAM, APHP-Sorbonne Universite, Pitie-Salpetriere Hospital, Paris, France; 2 GRC ARPE, Medecine Sorbonne-Universite, Paris, France; 3 Medecine Sorbonne-Université, Paris, France; 4 Réanimation Chirurgicale, CHU Pitié-Salpêtrière, 47-83 Boulevard de l’Hôpital, 75013 Paris, France

Dear editor,

We read with great interest the study by *Guillaume Franchineau* et al. [1], which assessed regional ventilation during prone positioning in acute respiratory distress syndrome (ARDS) patients treated with venovenous-extracorporeal membrane oxygenation (ECMO) using electrical impedance tomography (EIT). In this work, the authors found that patients with a compliance gain after prone positioning (defined as an increase in static respiratory system compliance of more than 3 mL/cmH_2_O) had a lower repartition of the tidal volume in the dorsal region of the lung at baseline. In these patients, compliance gain was associated with a reduction in PaCO_2_, despite constant sweep gas flow, suggesting a decrease in dead-space. Interestingly, this specific pattern of ventilation distribution evokes the one described in patients with focal ARDS. Indeed, different ARDS phenotypes have been defined using lung tomography [2]: focal or lobar ARDS with a loss of aeration in the lower and dorsal part of the lung while the upper lobes are not visually affected. And, in contrast, non-focal or diffuse ARDS in which loss of aeration is diffuse, affecting both ventral and dorsal regions. Strikingly, patients with focal ARDS respond to prone positioning in the same way as the patients exhibiting an increase in compliance during prone position in the Franchineau’s study. Although both focal and non-focal ARDS show improved oxygenation after prone positioning, only focal ARDS gain compliance and exhibit reduced PaCO_2_ [3]. As pointed out by *Franchineau* et al., the reduction of PaCO_2_ and not the increase of PaO_2_ has been linked to the beneficial impact of prone positioning. Thus, if prone positioning may benefit all ARDS patients, resulting in a more homogeneous distribution of aeration and reduced dead-space, its beneficial impact on survival is probably largely influenced by the predominance of the focal pattern among ARDS patients. This hypothesis was studied in the LIVE study which assessed whether a personalized mechanical ventilation tailored for lung morphology could improve ARDS survival outcomes compared to standard of care [4]. The results for the primary outcome were negative. However, misclassification of lung morphology occurred in 21% and excluding those misclassified patients in a *per*-*protocol* analysis led to a significant decrease in mortality in the personalized ventilation group.

Hence, lung morphology assessment seems to be a major issue. Using a single slice computed tomography to classify lung morphology seems to be inefficient according to LIVE results [4]. A dynamic lung tomography, defined as a two slices computed tomography (one obtained at 5 cm H_2_O and one at 45 cm H_2_O) to assess both lung morphology and the potential of lung recruitment could be of more value [5]. However, moving ARDS patients to CT-scan is not always feasible and safe, especially for patients treated with ECMO. A bedside and easy-to-use tool with real-time assessment is thus needed to personalized mechanical ventilation according to lung morphology. Some authors argue that lung ultrasonography may be of interest [5]. Lung ultrasound is an efficient bedside tool to assess lung morphology allowing to map the entire subpleural lung parenchyma. Moreover, similar to lung tomography, lung ultrasonography could be used to assess changes in lung morphology during a PEEP trial [5]. However, it does not allow to evaluate lung overdistension and only focuses on a restricted part of the subpleural lung parenchyma. EIT may be more effective, first to assess collapsed and overdistended regions and determine ARDS morphology, but also as a monitoring tool to assess response to therapies such as positive end expiratory pressure (PEEP) and prone positioning. The results of *Franchineau* et al. may be reconsidered as an additional clue of the usefulness of EIT in ARDS treatment. Rather than using EIT to monitor local compliance variation, EIT may be used to select prone position candidates with focal ARDS and to apply an optimal PEEP after a PEEP trial for non-focal patients.

To conclude, we believe that EIT may be used to assess lung morphology and apply personalized ventilation strategy. Further study comparing EIT with dynamic CT in order to characterize lung phenotype is thus needed. But first of all, it is mandatory to define what pattern or measure obtained from EIT could be used to allow a clear distinction of focal and non-focal ARDS. An example of such measurements, baseline Vt dorsal/Vt global as suggested by Franchineau’s study, could be of interest but needs to be prospectively studied and compared with other EIT-derived indices.

## Data Availability

Not applicable.

## Abbreviations

ARDS: Acute respiratory distress syndrome
ECMO: Extracorporeal membrane oxygenation
EIT: Electrical impedance tomography
PEEP: Positive end expiratory pressure
